# Retroviral RNA Dimerization and Packaging: The What, How, When, Where, and Why

**DOI:** 10.1371/journal.ppat.1001007

**Published:** 2010-10-07

**Authors:** Silas F. Johnson, Alice Telesnitsky

**Affiliations:** Department of Microbiology and Immunology, University of Michigan Medical School, Ann Arbor, Michigan, United States of America; University of California San Francisco, United States of America

## The Encapsidated Viral Genome: What Is Packaged?

**Figure 1:**
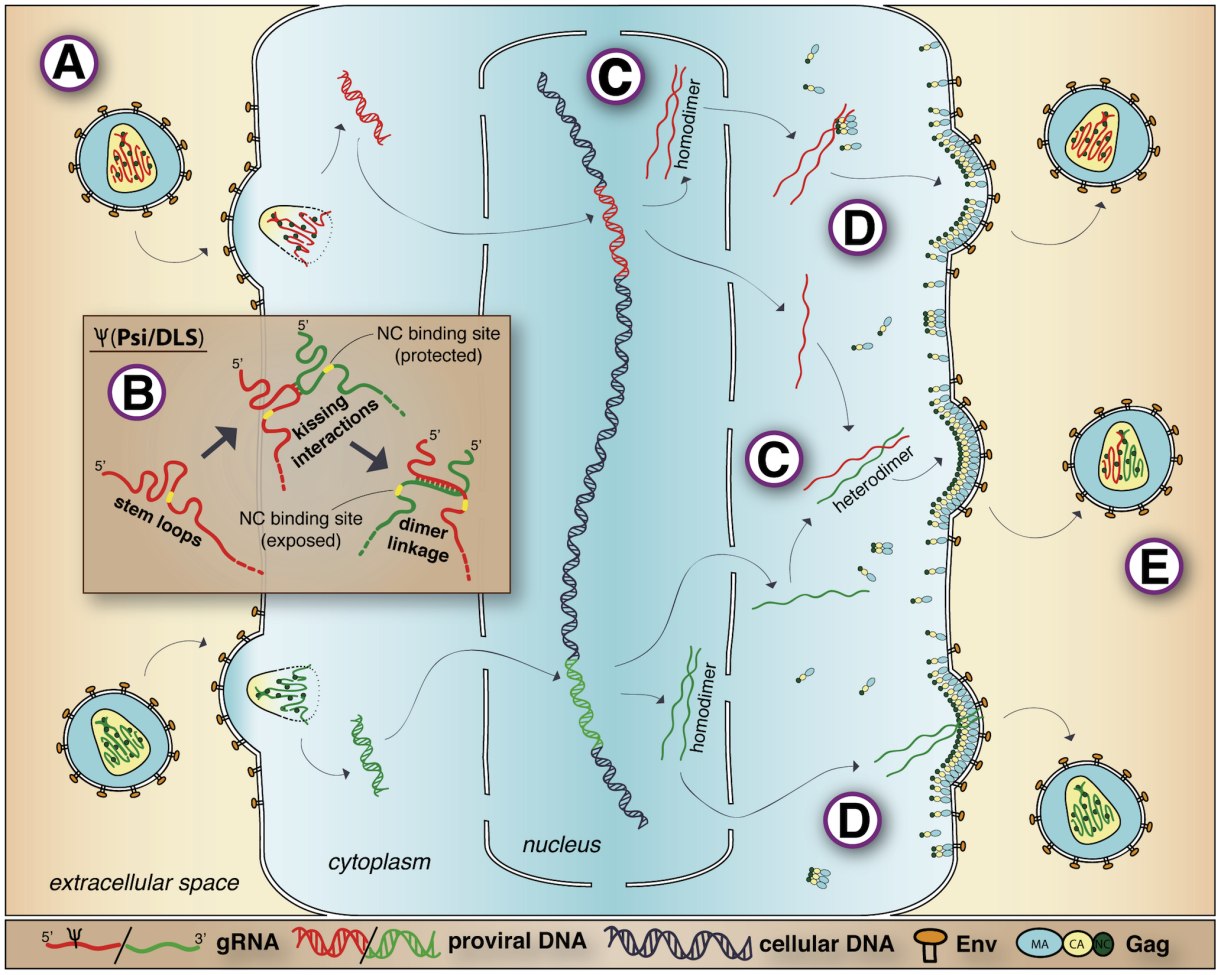
Retroviral RNA dimerization and packaging. The description of retrovirus genomic RNA dimerization and packaging is based on a representative co-infected cell and depicts properties of C-type retroviruses such as HIV-1 and MLV. Note that this figure represents concepts schematically and is not intended to accurately represent structures or scale. (A) *What is packaged:* two genetically complete but nicked copies of plus sense gRNA (shown in red at top or green at bottom) are packaged within the capsid core and joined by a dimer linkage. The co-packaged gRNAs are condensed in the core and bound by NC (shown as green circles). (B) *How gRNAs are recruited:* initial gRNA dimerization occurs via kissing interactions between palindromic stem loops. Subsequent basepairing register-shifts that occur during dimer linkage maturation expose single-stranded NC binding motifs (indicated in yellow) that were previously basepaired and thus sequestered in gRNA monomers, allowing for Gag binding during recruitment. (C) *When gRNAs associate in dimers:* the point at which RNA dimerization partners first associate is different for HIV-1 and MLV. MLV gRNA dimers first associate near sites of transcription in the nucleus, which leads to disproportionately large amounts of one homodimer or the other (shown in red at top and green at bottom). HIV-1 gRNA dimers first associate in the cytoplasm, leading to a random assortment of homodimeric and heterodimeric gRNAs. (D) *Where gRNAs join assembling virions:* gRNAs may form subassemblies with Gag in the cytoplasm (shown at top) or may associate at the plasma membrane (shown at bottom) after active transport separate from all or most Gags. (E) *Why two gRNAs are packaged:* the packaging of two gRNAs may aid in packaging specificity as well as the promotion of genomic integrity. In addition, the packaging of two genetically distinct gRNAs (shown as a red/green heterodimer) promotes genetic recombination, which leads to genetic diversity in viral progeny.

Retroviral genomic RNAs (gRNAs) are packaged as dimers, joined near their 5′ ends in non-covalent linkages that withstand modest heat treatment but dissociate at ∼65°C. Determinants of gRNA dimerization and recruitment for packaging map to the same ∼100 to ∼300 base regions and are, for the most part, physically and genetically inseparable [1]. Synthetic RNAs containing these sequences dimerize in vitro. Because transplanting these sequences onto a cell mRNA confers selective packaging, and ablating them greatly reduces gRNA packaging, these sequences are known as Ψ (psi), for “packaging signal” (Figure 1A) [2].

Within virions, gRNAs are coated with a basic viral protein called nucleocapsid (NC) at a density of about one NC per five to eight RNA bases [3]. This nucleoprotein complex resides within the mature virion core. Total gRNA length, were it in an A-helix, exceeds the core inner diameter by more than 30-fold [4]. Thus, encapsidated gRNA is highly condensed. The co-packaged gRNAs likely are not aligned along their lengths because they are identical and cannot basepair in register, but the nature of their compaction is unknown.

If Ψ is experimentally removed from gRNA but viral proteins are still expressed, morphologically normal virions can form, which are devoid of gRNA. These contain random samples of host mRNA [5]. Each Ψ+ or Ψ− virion also contains several copies of certain host RNAs such as 7SL, the RNA scaffold of signal recognition particles. Other than the primer tRNA, any roles of these host non-coding RNAs in retroviruses are unknown.

Usually, a virion's two gRNAs are identical. However, if a producer cell contains two distinct dimer-compatible proviruses, virions can contain gRNA heterdimers. Both gRNAs are genetically complete but not intact, as they appear to contain nicks and run as smears on denaturing gels. Accordingly, it has been speculated that retroviruses' dimeric genome organization may serve in part as defense against antiviral nucleases that would otherwise restrict replication [4]. RNA degradation during reverse transcription further limits provirus synthesis to one or fewer per virion [6]. Transmission of no more than one allele at each locus explains why, although they package two gRNAs, retroviruses are not truly diploid.

## How Are gRNA Dimers Recruited?

gRNA packaging specificity results from high-affinity interactions between NC and the dimer linkage structure (DLS) that forms when two gRNAs join [1] (Figure 1B). The NC: Ψ/DLS interaction differs from RNA coating by NC, both in its specificity and because the interaction occurs while NC is a domain of the Gag polyprotein. Changes to the NC coding region lead to deficiencies in gRNA packaging, and NC dictates packaging in chimeras where one retrovirus's NC is exchanged for another's [7].

Secondary structure and mutant analyses show that the Ψ/DLS region contains a series of RNA stem-loops. Single-stranded loops on some of these hairpins contain palindromes, allowing one gRNA to basepair via “kissing” interactions with a second gRNA (Figure 1B). This initial dimer contact is followed by structural transitions that extend intermolecular basepairing to form the encapsidated dimer linkage [1].

Despite 25 years of research and impressive advances in understanding secondary structure, we still lack a three-dimensional understanding of any virus's Ψ/DLS and what, minimally, is necessary for packaging. Conflicting reports may reflect complications in teasing apart elements in a multifunctional genome region. The propensity of retroviruses to package suboptimal RNAs in the absence of gRNAs, differences among retroviruses, and the absence of uniform naming conventions also cloud the picture.

The structural basis of why gRNAs are packaged as dimers is best understood for murine leukemia virus (MLV) [1]. MLV NC binds unpaired UCUG motifs with high affinity. These are overrepresented in MLV's 5′ untranslated region, but are basepaired and inaccessible to NC in monomeric gRNA. However, once dimerized, secondary structure register shifts expose the high affinity binding sites. Thus, discrimination between monomeric and dimeric MLV gRNAs appears based on UCUG availability in one of two alternate gRNA folds.

Ψ's presence is not always sufficient to specify packaging. The packaging machinery must discriminate between complete gRNAs and subgenomic mRNAs. For HIV-1 and gammaretroviruses, this discrimination reflects Ψ's location downstream of the 5′ splice site and its removal from subgenomic mRNAs. However, for retroviruses like HIV-2, major packaging elements are present on both spliced and unspliced RNAs. Avian leukosis virus (ALV) spliced RNAs also retain Ψ. Interestingly, a heterologous RNA containing ALV Ψ, but not the natural ALV Ψ+ *env* mRNA, is packaged well [8], which implies *env* mRNAs contain negative packaging elements.

## When Do the Two Co-Packaged gRNAs First Associate?

gRNAs are capped and polyadenylated unspliced RNA polymerase II transcripts, identical in sequence to *gag* mRNAs. All retroviruses that can mobilize virus gene-free retroviral vectors are by definition capable of at least some *trans*-packaging. However, whether an individual RNA can serve as both *gag* mRNA and gRNA varies among retroviruses.

All retroviruses must transport their unspliced RNAs to the cytoplasm, but retroviruses differ both in the RNA transport pathway used and in the replication step where mRNA and gRNA fates bifurcate [9]. Specifically, studies comparing gRNA and mRNA half-lives concluded both reside in a single, equilibrating pool for HIV-1, from which unspliced RNAs can be directed alternately into gRNA or *gag* mRNA fates well after transcription is completed [10]. In contrast, MLV gRNAs have shorter half-lives than mRNAs, and unspliced RNAs destined to serve gRNA roles appear to adopt this fate before exiting the nucleus [10], [11]. This suggests unspliced RNA fates are decided earlier for MLV than for HIV-1.

The timing of dimerization partner association is a slightly different question. By such criteria as their gel mobility, the gRNAs in immature virions can appear monomeric, and some have suggested monomeric gRNAs are the recruited species, with dimerization occurring later [12]. However, although means of recruiting gRNAs in a two-or-none fashion other than as immature dimers cannot be ruled out unambiguously, the preponderance of experimental evidence argues that initial gRNA dimer interactions occur prior to recruitment into assembling virions, with immature dimers matured upon protein processing. These observations include biases in gRNA co-packaging, the very poor packaging of gRNAs that are unable to dimerize, and the fact that even when gRNA is limiting and virions contain less than one gRNA on average, gRNAs are dimeric in those virions that contain them [13], [14].

The replication stage when dimerization partners first associate differs among retroviruses (Figure 1C). MLV gRNAs select dimerization partners at or near sites of transcription and proceed to assembly sites without releasing the partner they selected in the nucleus [15]. In contrast, HIV-1 gRNAs first associate for dimerization in the cytoplasm [16]. This trafficking difference has profound effects on virus genetics [4]. Retrovirus genomes are not segmented, but alleles re-assort at an exceptional rate due to high-frequency recombination, which results from template switching between dimerized gRNAs during reverse transcription. The timing of gRNA dimerization ensures that co-expressed HIV-1 gRNAs associate at random. In contrast, early self-associations of MLV gRNAs result in disproportionate co-packaging of identical RNAs, and template switching between identical gRNAs does not yield recombinants. As a result, genetic marker reassortment is about 10-fold lower for MLV than for HIV-1, even though these viruses' recombinogenic template switching rates are the same [4].

## Where Does the Packaging of gRNAs into Virions Initiate?

On the gross morphologic level, orthoretroviruses adopt one of two assembly pathways. Beta- and deltaretroviruses form electron-dense particles in the cytoplasm, while assembly for “C-type” viruses like HIV-1, MLV, and ALV is first detectable on membranes. Whether Gag and gRNA first come together at the plasma membrane or associate earlier is incompletely resolved, but recent findings suggest that at least for HIV-1, initial interactions occur at the plasma membrane [17] (Figure 1D).

gRNA's intracellular routes to assembly sites differ among viruses and are not well understood. gRNA transport likely exploits host directional trafficking machinery [18]. For Rous sarcoma virus, a subset of viral Gag transits back into the nucleus to recruit gRNA en route to the plasma membrane [19].

Dimer linkage structures are recruited via high affinity interactions with perhaps a dozen NC domains [14], [17]. This is less than 1% of the total Gag precursors that form a virion. What prevents the other Gags from engaging additional dimer linkages? Because virions form readily without any gRNA, and also when provided with gRNA three times the normal length, a phage-like RNA “headful” mechanism seems unlikely [20]. Recent work with co-expressed gRNAs, each tethered to a different fluorescent protein, confirmed that HIV-1 essentially always packages precisely one gRNA dimer [21]. Is some sort of RNA quorum sensing triggered once one DLS is engaged? And if so, what substitutes in Ψ− particles? Interestingly, certain RNA binding-defective NC mutants phenocopy properties associated with budding defects, suggesting interactions with gRNA may help drive a late assembly step [22], [23]. However, no detectable change is observed in the time required for assembly, whether or not particles contain gRNA [17].

## Why Do Retroviruses Package Dimeric gRNAs?

The fact that all other viruses encapsidate single copy genomes begs the question of why retroviruses co-package two gRNAs. One reason may be economy of scale: RNA dimerization allows formation of a unique structure—the dimer linkage—that distinguishes gRNAs from mRNAs. Monomeric HIV-1 RNAs are packaged when engineered with tandem dimer linkages, underscoring the importance of this RNA structure, and not gRNA counting per se, to packaging [24].

Co-packaging gRNAs allows retroviruses to generate intact proviruses despite pervasive gRNA nicking [4] (Figure 1E). Template switching during reverse transcription is probably why retroviruses maintain infectivity when their gRNAs are damaged by gamma rays, and why retroviruses are much less radiation-sensitive than RNA viruses like vescicular stomatitis virus [25]. Researchers previously thought retroviral recombination might be mutagenic, but these notions have been dispelled [4]. HIV-1 particles with two gRNAs generate full-length proviruses more efficiently than virions engineered to contain single gRNAs [26]. Thus, another advantage of gRNA dimers appears to be increased replication fidelity.

Co-packaging gRNAs promotes higher recombination frequencies for retroviruses than all other viruses, allowing rapid loss of deleterious alleles and re-assortment of genome segments. With approximately three to ten crossovers occurring during the synthesis of every provirus, recombination is perhaps 10-fold more frequent than reverse transcriptase base substitution rates, and is an evolutionary driving force for retroviruses such as HIV-1 that display high levels of replication and multi-strain infection [4].

These observations help seal the case for likely evolutionary advantages of dimeric genome packaging. The DLS in its immature form, which results only upon association of two gRNAs, likely provides the means for selective gRNA packaging. The need to generate an intact provirus provides a strong motive for packaging redundant genetic information. And because retroviruses encapsidate gRNA dimers, recombination can provide the opportunity for almost limitless combinatorial genetic sampling.
